# Propane Steam Reforming over Catalysts Derived from Noble Metal (Ru, Rh)-Substituted LaNiO_3_ and La_0.8_Sr_0.2_NiO_3_ Perovskite Precursors

**DOI:** 10.3390/nano11081931

**Published:** 2021-07-27

**Authors:** Theodora Ramantani, Georgios Bampos, Andreas Vavatsikos, Georgios Vatskalis, Dimitris I. Kondarides

**Affiliations:** Department of Chemical Engineering, University of Patras, 26504 Patras, Greece; ramantani@chemeng.upatras.gr (T.R.); geoba@chemeng.upatras.gr (G.B.); up1019056@upnet.gr (A.V.); up1047645@upnet.gr (G.V.)

**Keywords:** propane, steam reforming, hydrogen production, perovskite, ruthenium, rhodium, La_2_O_2_CO_3_, stability

## Abstract

The propane steam reforming (PSR) reaction was investigated over catalysts derived from LaNiO_3_ (LN), La_0.8_Sr_0.2_NiO_3_ (LSN), and noble metal-substituted LNM*_x_* and LSNM*_x_* (M = Ru, Rh; *x* = 0.01, 0.1) perovskites. The incorporation of foreign cations in the A and/or B sites of the perovskite structure resulted in an increase in the specific surface area, a shift of XRD lines toward lower diffraction angles, and a decrease of the mean primary crystallite size of the parent material. Exposure of the as-prepared samples to reaction conditions resulted in the in situ development of new phases including metallic Ni and La_2_O_2_CO_3_, which participate actively in the PSR reaction. The LN-derived catalyst exhibited higher activity compared to LSN, and its performance for the title reaction did not change appreciably following partial substitution of Ru for Ni. In contrast, incorporation of Ru and, especially, Rh in the LSN perovskite matrix resulted in the development of catalysts with significantly enhanced catalytic performance, which improved by increasing the noble metal content. The best results were obtained for the LSNRh_0.1_-derived sample, which exhibited excellent long-term stability for 40 hours on stream as well as high propane conversion (X_C3H8_ = 92%) and H_2_ selectivity (S_H2_ = 97%) at 600 °C.

## 1. Introduction

The continuously increasing energy demand associated with global population growth and the rapid evolution of the industrial sectors has led to the search for alternative energy systems that are affordable, reliable, and sustainable, with low environmental impact [1,2]. Hydrogen (H_2_) appears to be the most promising future energy carrier since, when combined with fuel cells, it makes it possible to produce electricity for mobile, stationary, and industrial applications with minimal pollutant emissions [3,4,5]. Hydrogen is mainly produced via hydrocarbon conversion employing steam reforming (SR), dry reforming (DR), autothermal reforming (ATR), and partial oxidation (POX) reactions [6]. Currently, the most common industrial process for hydrogen production is the steam reforming of natural gas, which accounts for ca. 85% of the total H_2_ produced worldwide [3,4,7]. Other fuels that can be used for this purpose include ethanol, methanol, gasoline, ammonia, and dimethyl ether [8,9] as well as light alkanes such as propane and butane [10,11,12]. The use of propane as a source of hydrogen has attracted significant attention in recent years because of its favorable physical properties (liquefaction at room temperature and ca. 9 bar), which facilitate its safe handling, storage, and transportation as well as its relatively low cost, abundance, and availability through the existing distribution network of liquefied petroleum gas (LPG) [13,14,15,16,17].

Propane steam reforming (PSR) is a strongly endothermic reaction (1) requiring high temperatures (>700 °C) to succeed high H_2_ yields, and proceeds in parallel with the moderately exothermic water-gas shift (WGS) reaction (2):

C_3_H_8_ + 3 H_2_O ↔ 3 CO + 7 H_2_         ΔH° = 499 kJ mol^−1^ (1)

CO + H_2_O ↔ CO_2_ + H_2_         ΔH° = −41 kJ mol^−1^ (2)

By-products such as methane and ethylene may also be produced under reaction conditions via the CO/CO_2_ methanation [18] and propane decomposition reactions [19], respectively. The PSR reaction can be carried out by employing Ni-based catalysts, which have been extensively studied because of the good activity and low cost of nickel, compared to precious metals [19]. However, the application of high temperatures results in the sintering of Ni particles and the concomitant deterioration of catalytic performance [20,21]. In addition, exposure of Ni-based catalysts to reforming reaction conditions results in carbon deposition on their surface via the Boudouard reaction or/and the decomposition of propane and by-product hydrocarbons such as methane and ethylene, which further accelerate the catalyst deactivation [11,15,22,23,24,25]. Consequently, research efforts currently focus on the development of novel catalytic systems, with improved resistance against coke deposition and metal particle agglomeration [21]. In this respect, noble metals such as Rh, Ru, Pt, and Ir have been studied either alone or in combination with Ni because of their lower tendency to form coke [14,26,27,28,29,30]. The addition of promoters such as alkali metals in the form of oxides has also been shown to suppress coke deposition, but results in generally inferior catalytic activity [10,22].

Recently, perovskite-derived catalysts have attracted significant attention for the production of H_2_ or syngas via reforming reactions [3] because of their high activity, carbon tolerance, thermal stability, and low cost [23,31]. Perovskites are a class of crystalline oxides described by the general formula ABO_3_, where the A-site (12-fold coordination) is generally occupied by an alkaline-earth or alkali metal cation with a larger size, and the B-site (6-fold coordination) is occupied by a transition metal ion with a smaller radius [3,32,33]. Generally, the A-sites provide the basicity and high thermal stability of the whole structure. When perovskites are exposed to a reductive environment, the B-site cations migrate (exsolve) spontaneously from the bulk to the surface, forming highly dispersed metal nanoparticles. The exsolved metal particles, which are confined on the catalyst surface, exhibit superior reactivity and stability due to their enhanced resistance to coking and metal sintering [32,33]. Removal of the active metal from the host lattice results in the rearrangement of the nonreducible metals and the formation of oxygen vacancies [32]. The physicochemical and catalytic properties of perovskite-derived catalysts can be greatly influenced by the partial substitution of the A- and/or B-sites by other metal cations, which results in the formation of solids described by the formula A_1−*x*_Á*_x_*B_1−*y*_B́*_y_*O_3_ [31,34]. As a general trend, A-site substitution enhances the oxygen mobility in the perovskite structure by generating oxygen vacancies, which suppress the carbon deposition, while B-site substitution tends to increase the activity and stability of the derived catalysts due to bimetallic synergy effects [3,33].

The performance of perovskite-derived catalysts for hydrogen production has been studied by several authors. The majority of these studies focused on the dry reforming of methane [3,35,36,37,38,39,40,41,42,43] and to a lesser extent on the reformation of other feedstocks such as acetic acid [44,45], biomass tar [46], bio-oil [47], diesel [48,49,50], ethane [51], ethanol [52,53,54], glycerol [55,56], phenol [57], and toluene [58,59]. The propane reforming over perovskite-derived catalysts has rarely been reported in the literature [60,61,62]. For example, Lim et al. [60] investigated the autothermal reforming of propane over Ce-modified Ni/LaAlO_3_ catalysts and reported the increased thermal stability and low carbon deposition over the Ce-doped sample. Yeyongchaiwat et al. [62] studied the oxidative reforming of C_3_H_8_ over Pr_2_Ni_0.75_Cu_0.25_Ga_0.05_O_4_ perovskite whereas Sudhakaran et al. [61] investigated the dry reforming of propane over the SrNiO_3_ catalyst. The observed enhancement of CO selectivity and minimal carbon formation was attributed to the strong basicity of the perovskite [61]. 

In the present work, the propane steam reforming reaction was investigated over catalysts derived from LaNiO_3_-based perovskites promoted with Sr and/or noble metals (Ru, Rh). The LaNiO_3_ perovskite was chosen as the parent material because of its well-known activity and stability for reforming reactions [3,63] originating from its transformation into metallic nickel (Ni^0^) and lanthanum oxide (La_2_O_3_) when exposed to reductive conditions at high temperatures [31]. The effects of partial substitution of La by Sr in the A-sites and/or of Ni by Ru or Rh in the B-sites of LaNiO_3_ have been investigated in an attempt to systematically study the effects of the nature and composition of the A- and B-sites on the catalytic performance for the title reaction. Strontium was chosen because of its ability to increase the number of vacancies that facilitates the mobility of oxygen toward the surface of the solid, to improve the adsorption and desorption of CO_2_, and to enhance the resistance of the derived catalysts against carbon deposition [64,65,66]. On the other hand, partial substitution of Ni by small amounts of Ru or Rh aimed at further improving the catalytic activity and resistance against deactivation, because of the excellent affinity of these metals for the rupture of C–C and C–H bonds and their ability to minimize coke accumulation [32,52,67,68]. Results obtained show that catalysts derived from LSNRu*_x_* and, especially, LSNRh*_x_* perovskites are characterized by high activity and selectivity toward H_2_ as well as by long-term stability for the PSR reaction. It is anticipated that the present work may contribute toward understanding the effects of the nature and composition of the A and B sites on the catalytic performance of perovskite-derived catalysts, thereby facilitating the design of efficient catalytic materials for the steam reforming of propane and other similar reactions.

## 2. Materials and Methods

### 2.1. Synthesis of Perovskite-Type Oxides

A series of perovskites including LaNiO_3_ (LN), La_0.8_Sr_0.2_NiO_3_ (LSN), and noble metal-substituted oxides denoted in the following as LNM*_x_* and LSNM*_x_* (M = Rh or Rh; *x* = 0.01 or 0.1) were prepared with the combustion synthesis method [69]. Citric acid monohydrate (Merck, Darmstadt, Germany) was used as a fuel whereas Sr(NO_3_)_2_ (Sigma-Aldrich, St. Louis, MO, USA), La(NO_3_)_3_·6H_2_O (Alfa Aesar, Karlsruhe, Germany), Ni(NO_3_)_2_·6H_2_O (Alfa Aesar, Karlsruhe, Germany), Ru(NO)(NO_3_)_3_ (Alfa Aesar, Karlsruhe, Germany), and N_3_O_9_Rh (Alfa Aesar, Karlsruhe, Germany) were used as metal precursor salts. In a typical synthesis, stoichiometric quantities of the respective metal nitrates were dissolved in triple-distilled water followed by the addition of citric acid, NH_4_NO_3_ (extra-oxidant), and, finally, ammonia solution for the neutralization of the excess citric acid. The resulting solution was heated until ignition at ca. 400 °C and the powder obtained was calcined at 900 °C for 5 h and then ground in a mortar. The notations and nominal formulas of the materials thus prepared are listed in Table 1.

### 2.2. Physicochemical Characterization

The specific surface areas (SSAs) of the freshly prepared and the used materials were determined with the BET method using a Micromeritics (Gemini III 2375) instrument (Norcross, GA, USA). X-ray diffraction (XRD) patterns were obtained on a Bruker D8 instrument (Bruker, Karlsruhe, Germany) and were analyzed by employing JCPDS data files. The average crystallite size of the resulting phases was estimated by applying the Scherrer equation. Details on the above methods and procedures can be found elsewhere [70]. Scanning electron microscopy (SEM) images were obtained on the JEOL JSM 6300 instrument (JEOL, Akishima, Tokyo, Japan), equipped with X-ray Energy Dispersive Spectrometer, EDS (ISIS Link 300, Oxford Instruments, Oxford, UK).

### 2.3. Catalytic Performance Tests

The catalytic performance of the synthesized materials for the propane steam reforming reaction was evaluated using a fixed bed reactor operating at near atmospheric pressure. The reactor consists of a 35-cm long quartz tube (6 mm O.D.) with an expanded 6-cm long section in the middle (12 mm O.D.) where the catalyst was placed. The flow of the inlet gases is controlled by employing mass-flow controllers (MKS Instruments, Andover, MA, USA). An HPLC pump (type Marathon; Spark-Holland, Emmen, The Netherlands) was used to feed water into a vaporizer with a set temperature of 200 °C and the steam produced was mixed with the gas stream coming from the mass-flow controllers. The reaction temperature was measured in the middle of the catalyst bed using a K-type thermocouple placed within a quartz capillary well running through the cell. The reactor is placed in an electric furnace, the temperature of which is controlled using a second K-type thermocouple placed between the reactor and the walls of the furnace. A pressure indicator was used to measure the pressure drop in the catalyst bed. A cold trap was placed at the exit of the reactor to condense water before the introduction of the sample to the analysis system. Separation and analysis of hydrocarbons such as C_3_H_8_, C_3_H_6_, C_2_H_6_, C_2_H_4_, and CH_4_ was accomplished with a gas chromatograph (GC-14A, Shimadzu, Kyoto, Japan) equipped with a Carbosieve column and an FID detector, whereas analysis of Ar, CO, CO_2_, and CH_4_ was made using a Carboxen column and a TCD detector using He as the carrier gas. A separate GC-TCD system (GC-8A, Shimadzu, Kyoto, Japan), operated with N_2_ as the carrier gas, was used to measure the amount of produced H_2_. Determination of the response factors of the GC detectors was performed using gas streams of known composition.

In a typical experiment, 100 mg of a fresh perovskite sample (particle size: 0.18 mm < *d* < 0.25 mm) was placed in the reactor and heated at 750 °C under He flow. The flow was then switched to the reaction mixture (200 cm^3^ min^−1^) consisting of 2.3% C_3_H_8_, 22.9% H_2_O, and 0.7% Ar, which was used as an internal standard (He balance). The reactor effluent was analyzed using the gas chromatographs described above. The reaction temperature was then stepwise decreased down to 450 °C and similar measurements were obtained. The propane conversion was calculated using the following equation

(3) $$X_{C3H8}=\frac{{\left[C_{3}H_{8}\right]}_{\mathrm{in}}-{\left[C_{3}H_{8}\right]}_{\mathrm{out}}}{{\left[C_{3}H_{8}\right]}_{\mathrm{in}}}\times 100$$

where [C_3_H_8_]_in_ and [C_3_H_8_]_out_ are the inlet and outlet concentrations of C_3_H_8_, respectively. 

The selectivities toward carbon-containing products (e.g., CO, CO_2_, CH_4_, C_2_H_4_, C_2_H_6_) were evaluated according to:

(4) $$S_{i}=\frac{\left[C_{i}\right]\times n}{\sum \left[C_{i}\right]\times n}\times 100$$

where *n* is the number of carbon atoms in product *i* and *C*_*i*_ is its concentration at the reactor effluent. Hydrogen selectivity is defined as the concentration of H_2_ in the effluent gas over the sum of the concentrations of all reaction products containing hydrogen, multiplied by the number of hydrogen atoms (*y*) in each product:

(5) SH2=[H2][H2]+∑(y2×[CxHy]i)×100

### 2.4. Temperature-Programmed Oxidation Experiments

The amount of carbon deposits accumulated on the catalyst surface following exposure to reaction conditions was estimated with temperature-programmed oxidation (TPO) experiments employing the apparatus and following the procedure described elsewhere [71]. Briefly, the “used” catalyst sample was placed in a quartz microreactor, exposed to a 3% O_2_/He mixture at room temperature for 10 min, and then heated linearly (*β* = 10 °C min^−1^) up to 750 °C under the same flow. Analysis of gases at the reactor effluent was accomplished by employing an online mass spectrometer (Omnistar/Pfeiffer Vacuum). The transient-MS signals at *m*/*z* = 2 (H_2_), 18 (H_2_O), 28 (CO), 32 (O_2_), and 44 (CO_2_) were continuously recorded. Responses of the mass spectrometer were calibrated against self-prepared mixtures of accurately known composition.

## 3. Results and Discussion

### 3.1. Physicochemical Characteristics of the as-Prepared Perovskite Samples

The specific surface areas of the as-prepared (fresh) perovskite samples were measured with the BET method and the results obtained are listed in Table 1. It was observed that the SSA of LaNiO_3_ (LN) was very low (3 m^2^ g^−1^). This is typical for perovskite-type oxide structures where SSA corresponds mainly to the external area of non-porous particles [72]. Partial substitution of La by Sr resulted in an increase of the SSA to 5 m^2^ g^−1^ for the La_0.8_Sr_0.2_NiO_3_ (LSN) sample. This is in agreement with the results of previous studies, which showed that partial substitution of the A-sites of a perovskite generally results in an increase of the SSA induced by structural disorder and delay in the crystallite growth [52,73,74]. A further increase of the SSA up to 8 m^2^ g^−1^ was observed upon partial substitution of Ni by Ru or Rh in the B-sites of the LN and LSN perovskites, which is more significant for the samples containing larger amounts of noble metals (Table 1).

**Figure 1:**
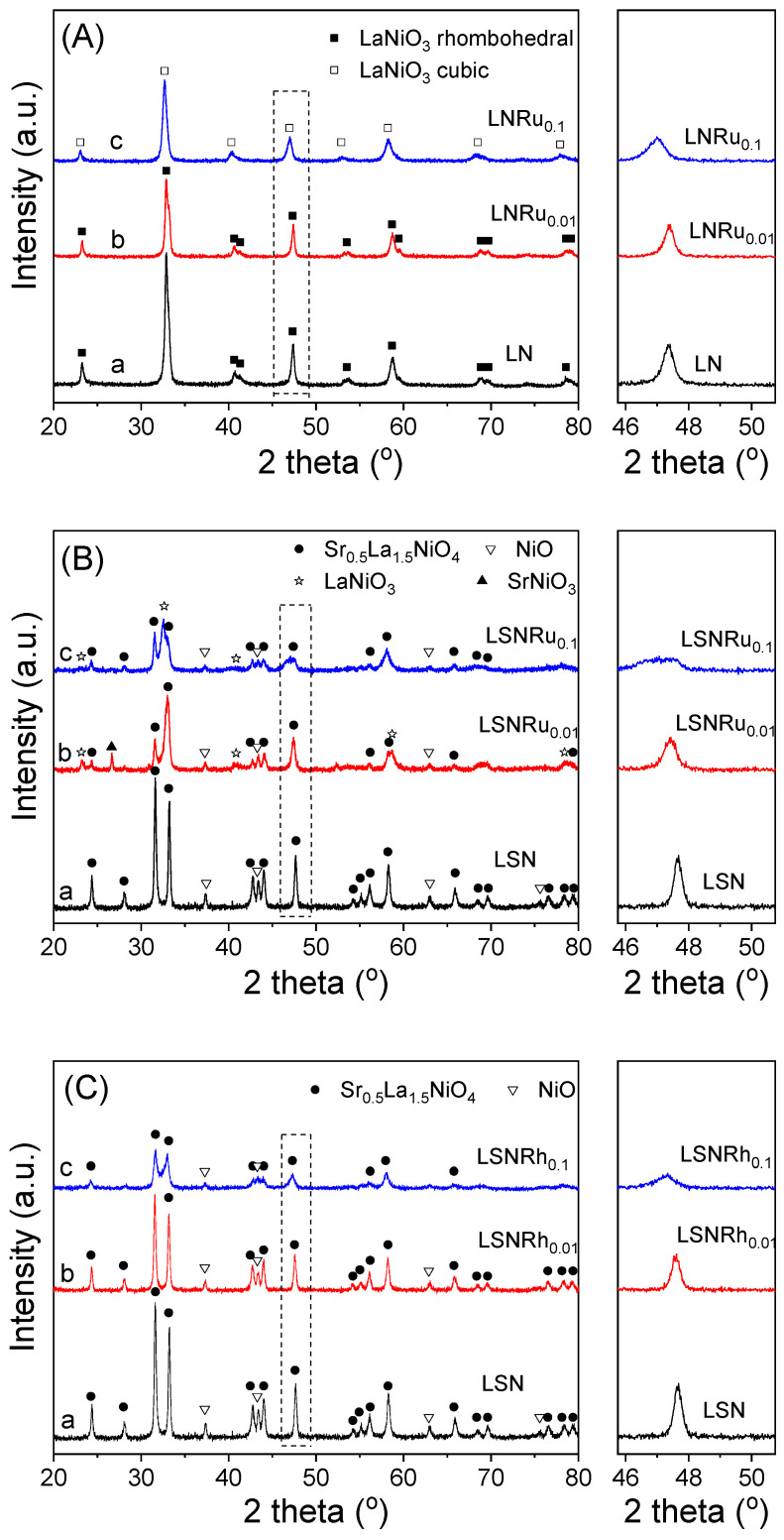
X-ray diffraction patterns of the as-prepared perovskite samples: (**A**) LNRu*_x_*, (**B**) LSNRu*_x_*, and (**C**) LSNRh*_x_*. In the magnified pattern on the right of each graph is shown the peak attributed to the (200) reflection.

The XRD patterns of the freshly prepared samples are shown in Figure 1, and the various phases detected in each case are listed in Table 1. It was observed that the diffractogram obtained for LN (Figure 1A, trace a) contained only reflections attributable to the rhombohedral LaNiO_3_ perovskite structure (JCPDS Card No. 34-1028). Substitution of 1% at. of Ni by Ru in the B-sites of LN (LNRu_0.01_ sample) resulted in a shift of the XRD peaks toward lower angles (trace b). This shift, which becomes more pronounced upon further increase of the Ru content (LNRu_0.1_ sample, trace c), indicates the preservation of the LaNiO_3_ perovskite structure but in the cubic system (JCPDS Card No. 33-710).

The sample obtained following partial substitution of 1% at. Ni by Ru in the LSN structure (LSNRu_0.01_ sample) was also characterized by the presence of Sr_0.5_La_1.5_NiO_4_ and NiO phases, whereas smaller peaks due to LaNiO_3_ (cubic) and SrNiO_3_ (hexagonal) could also be observed (trace b). Increasing the Ru content resulted in the formation of the same phases except for SrNiO_3_, which did not appear in the XRD pattern of the LSNRu_0.1_ sample (trace c). The presence of the NiO phase in the diffraction patterns of all LSNRu*_x_* samples (Figure 1B) indicates that a portion of nickel is located outside the perovskite structure. Regarding the LSNRh*_x_* samples, the XRD patterns presented in Figure 1C show that the partial substitution of Ni by Rh in the B-sites of the perovskite resulted in the formation of the same main phases observed for LSN (Sr_0.5_La_1.5_NiO_4_ and NiO). The peaks attributed to Sr_0.5_La_1.5_NiO_4_ were much broader for the LSNRh_0.1_ sample (trace c), compared to LSNRh_0.01_ (trace b), indicating the development of smaller crystallites. The calculated cell parameters of the crystalline phases detected by XRD for the as-prepared perovskite samples are presented in Table S1. It was observed that the incorporation of Ru or Rh in the LSN samples resulted in an increase in the *a* cell parameter and a decrease in the *c* cell parameter of the Sr_0.5_La_1.5_NiO_4_ tetragonal structure.

It is of interest to note that the XRD patterns presented in Figure 1 do not contain any peaks attributable to Ru or Rh species, indicating that noble metals are incorporated in the perovskite structure. It is known that the formation of perovskites may be restricted depending on the size of the cations present in the A and/or B sites of the material. In general, the radius of cation A should be larger than 0.09 nm, whereas the radius of cation B should be larger than 0.051 nm [75]. The partial substitution of A by an alkaline earth metal Á and/or the partial substitution of B by another transition metal B́ may result in the destruction of the perovskite matrix [75,76]. This can be avoided if the radii of A (*r*_A_), B (*r*_B_), and oxygen (*r*_O_) ions in the perovskite structure obey the restriction 0.75 < *t* < 1.0, where *t* is the tolerance factor expressed by [75]:

(6) $$t=\frac{\left(r_{A}+r_{O}\right)}{\sqrt{2}\left(r_{B}+r_{O}\right)}$$

Based on the above, and considering the sizes of the Ru and Rh cations, it may be concluded that the noble metals used as promoters in this work can only be incorporated in the B-sites of the LN and LSN perovskite structures.

**Figure 2:**
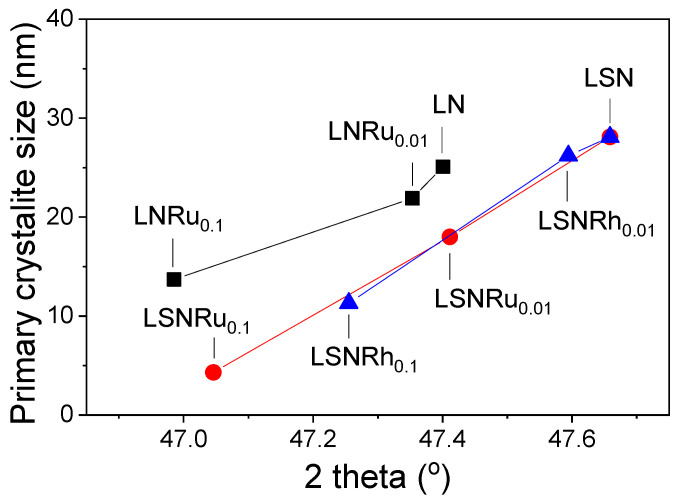
Dependence of the mean primary size of the perovskite crystallites, and of the angle corresponding to the (200) reflection on the type (Ru or Rh) and content (*x* = 0, 0.01, 0.1) of the B-site cation in LNRu*_x_*, LSNRu*_x_*, and LSNRh*_x_* samples (data extracted from the XRD patterns shown in Figure 1).

As already mentioned, the partial substitution of Ni by Ru or Rh resulted in a shift in the XRD reflections of the LN and LSN perovskites toward lower angles (2*θ*), which is accompanied by a decrease in the mean primary size of the perovskite crystallites, estimated by the Scherrer equation. This was the case, for example, for the (200) reflection which can be clearly discerned in the XRD patterns of all samples investigated (see expanded sections of the XRD patterns in Figure 1). The dependence of the (200) diffraction angle (2*θ*) and the mean crystallite size on the type (Ru or Rh) and content (*x* = 0, 0.01, 0.1) of the noble metal in the LNRu*_x_*, LSNRu*_x_*, and LSNRh*_x_* samples are shown in Figure 2. It was observed that in all cases, partial substitution of Ni by Ru or Rh resulted in a progressive shift of the diffraction peak toward lower angles and in the development of smaller crystallites. Qualitatively similar results have been reported by other authors [48,69,77]. For example, Mota et al. [48] found that substitution of Ru for Co in LaCo_1−*x*_Ru*_x_*O_3_ resulted in a shift of XRD peaks toward lower 2*θ* values accompanied by an alteration in the perovskite structure from rhombohedral to orthorhombic. This has been attributed to the larger ionic radius of Ru^3+^ (0.68 Å) compared to Co^3+^ (0.61 Å) and the concomitant decrease of the tolerance factor. Similar results were obtained by Ivanova et al. [77] over LaNi*_x_*Co_1−*x*_O_3_ and LaFe*_x_*Co_1−*x*_O_3_ perovskites as well as by Safakas et al. [69], who also observed a change in the perovskite structure from orthorhombic to rhombohedral upon partial substitution of Fe by Co in La_0.8_Sr_0.2_Co_x_Fe_1−*x*_O_3−*δ*_. In the present work, a change in the LaNiO_3_ structure from rhombohedral to cubic was observed following partial substitution of Ni by Ru (Figure 1A). The shift toward lower 2*θ* angles observed for the LNRu*_x_*, LSNRu*_x_*, and LSNRh*_x_* samples (Figure 2) indicates a high degree of incorporation of the noble metals in the B-sites of the perovskite structure [49] and can be explained considering that the ionic radii of Ru^3+^ (0.68 Å) and Rh^3+^ (0.67 Å) are larger than that of Ni^3+^ (0.60 Å).

Regarding the decrease in the primary crystallite size of the perovskites following substitution of Ni by Ru or Rh (Figure 2), it can be attributed to structural disorder and delay in the crystallite growth above-mentioned, and is reflected to the higher SSA of the noble metal-containing samples (Table 1). Qualitatively similar results have been reported by Mota et al. [48,49] for LaCo_1−*x*_Ru*_x_*O_3_ perovskites.

A representative SEM image obtained for the fresh LSNRh_0.1_ perovskite sample is shown in Figure S1, together with elemental mapping results, which confirm the presence and uniform distribution of La, Sr, Ni, and Rh in the material. Moreover, the elemental analysis showed that the wt.% content of La (47.42%), Sr (7.34%), Ni (22.04%), and Rh (4.16%) is in agreement with the nominal composition of this sample.

### 3.2. Catalytic Performance Tests

**Figure 3:**
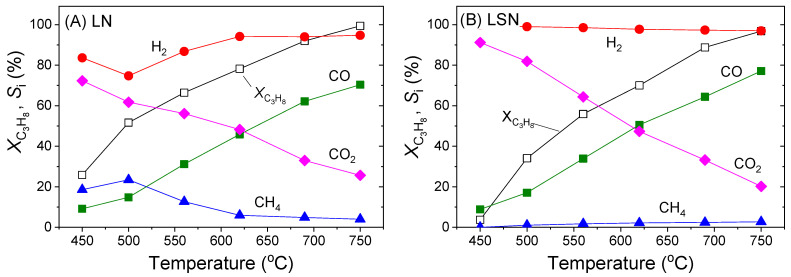
Conversion of propane (XC_3_H_8_) and selectivities to the indicated reaction products obtained over (**A**) the LaNiO_3_ (LN) and (**B**) the La_0.8_Sr_0.2_NiO_3_ (LSN) samples. Experimental conditions: mass of catalyst: 100 mg; particle diameter: 0.18 < d < 0.25 mm; feed composition: 2.3% C_3_H_8_, 22.9% H_2_O, 0.7% Ar (balance He); total flow rate: 200 cm^3^ min^−1^.

Results of catalytic performance tests performed under PSR reaction conditions using the noble metal-free LN and LSN perovskite samples are shown in Figure 3, where the conversion of propane (*X*_C3H8_) and the selectivities to reaction products (*S_i_*) are plotted as functions of reaction temperature. It was observed that the LN sample exhibited considerable activity for the title reaction, with *X*_C3H8_ increasing progressively from 26% at 450 °C to ca. 100% at 750 °C (Figure 3A). The selectivity toward CO_2_ decreased with increasing temperature while *S*_CO_ followed the opposite trend, indicating the occurrence of the reverse water gas shift (RWGS) reaction (reverse of Equation (2)), which is thermodynamically favored at higher temperatures [78].

Production of H_2_ exceeds 95% at temperatures higher than 550 °C. However, at lower temperatures, *S*_H2_ is decreased due to the production of methane. Selectivity to methane passes through a maximum of ca. 20% at around 500 °C and progressively decreases with increasing temperature due to the onset of the CH_4_ steam reforming reaction. In addition to the above-mentioned products, negligible amounts of C_2_H_4_ and C_2_H_6_ were also detected at the reactor effluent (not shown for clarity). The propane conversion curve obtained for the LSN sample (Figure 3B) shifted toward higher temperatures, compared to LN, indicating that the partial substitution of La with Sr in the A-sites of the perovskite negatively affects the catalytic activity. This is in general agreement with results of previous studies showing that substitution of Sr into the A-sites of LaNiO_3_ [64,79] or LaCoO_3_ [80] perovskites usually decreases the CH_4_ reforming activity. On the other hand, LSN is highly selective toward H_2_, with *S*_H2_ exceeding 97% over the entire temperature range investigated (Figure 3B). 

**Figure 4:**
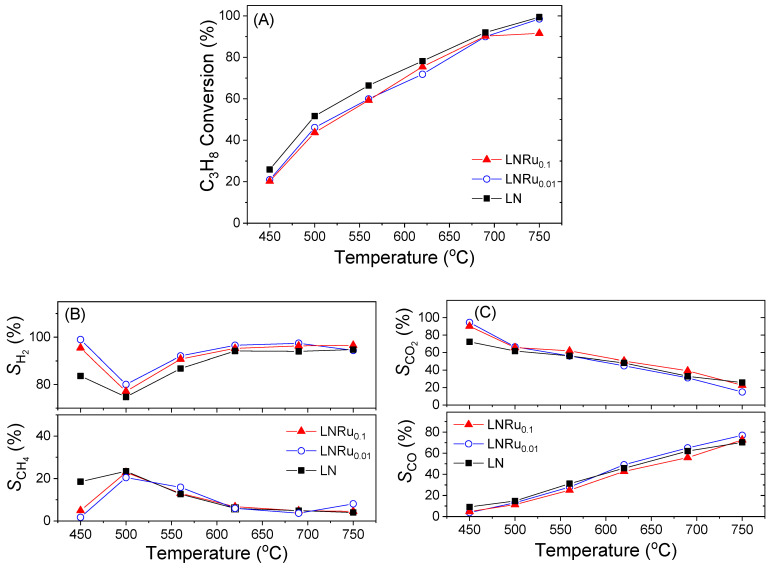
(**A**) Conversion of propane, (**B**) selectivities to H_2_ and CH_4_, and (**C**) selectivities to CO_2_ and CO obtained over the LNRu*_x_* catalysts. Experimental conditions: same as in Figure 3.

Results of similar catalytic performance tests obtained for the Ru-substituted LaNiO_3_ perovskites (LNRu_0.01_ and LNRu_0.1_ samples) are shown in Figure 4. It was observed that partial substitution of Ni by Ru resulted in slightly lower propane conversions compared to unpromoted LN (Figure 4A). This can be attributed, at least in part, to the different crystal structures obtained following the addition of Ru (i.e., cubic for LNRu_0.1_ vs. rhombohedral for LN (see Figure 1A)). Regarding selectivities to reaction products, the results presented in Figure 4B,C show that the presence of Ru does not appreciably affect the product distribution.

**Figure 5:**
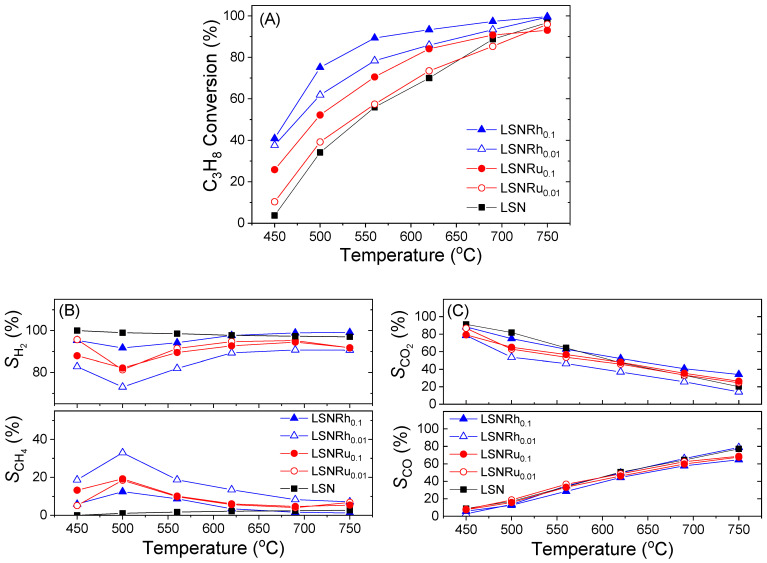
(**A**) Conversion of propane, (**B**) selectivities to H_2_ and CH_4_, and (**C**) selectivities to CO_2_ and CO obtained over the LSNRh_x_ and LSNRu_x_ catalysts. Experimental conditions: same as in Figure 3.

Results obtained following the substitution of Ni by Ru or Rh in the B-sites of LSN are presented in Figure 5. Regarding the sample with the lower Ru content (LSNRu_0.01_), it was observed that the propane conversion curve was slightly improved compared to pristine LSN (Figure 5A). An increase in the Ru content (LSNRu_0.1_ sample) resulted in a moderate increase in propane conversion in the whole temperature range investigated. Regarding selectivities to reaction products, both the LSNRu_0.01_ and LSNRu_0.1_ catalysts yielded higher amounts of methane compared to LSN (Figure 5B), whereas *S*_CO_ and *S*_CO2_ were not significantly affected (Figure 5C). On the other hand, the substitution of Ni by a small amount of Rh (1% at.) in the B-sites of the LSN perovskite (LSNRh_0.01_ sample) resulted in a significant increase of propane conversion in the whole temperature range investigated, compared to the pristine LSN (Figure 5A). For example, *X*_C3H8_ increased from ca. 4% to 38% at 450 °C and from 70 to 86% at 620 °C. This was accompanied by a decrease in the selectivity toward H_2_ and the production of larger amounts of CH_4_, which was maximized at ca. 550 °C (Figure 5B). The selectivity toward CO_2_ followed the opposite trend than that of CH_4_, whereas *S*_CO_ was not appreciably affected by the presence of Rh (Figure 5C). Further increase in the Rh content (LSNRh_0.1_ sample) resulted in an even higher propane conversion, which exceeded 90% at temperatures above ca. 550 °C (Figure 5A). Regarding selectivity to reaction products, the LSNRh_0.1_ sample exhibited superior selectivity toward H_2_, with *S*_H2_ being higher than 91% over the entire temperature range investigated and close to 100% above ca. 620 °C (Figure 5B). The selectivity toward CO_2_ was also very high and comparable to that of LSN (Figure 5C).

### 3.3. Physicochemical Characteristics of the Used Catalyst Samples

The “used” catalyst samples obtained following the catalytic performance tests presented in Figure 5 were characterized with the use of BET and XRD techniques in order to investigate the effects of exposure to the reaction conditions on the physicochemical properties of the materials. 

As shown in Table 1, the SSAs of the used samples were considerably larger than those of the fresh perovskites. For example, the SSA of the LNRu_0.1_ sample increased from 6 m^2^ g^−1^ to 71 m^2^ g^−1^. As discussed below, this increase in SSA was due to the destruction of the perovskite structures and the formation of new phases under reaction conditions. 

**Figure 6:**
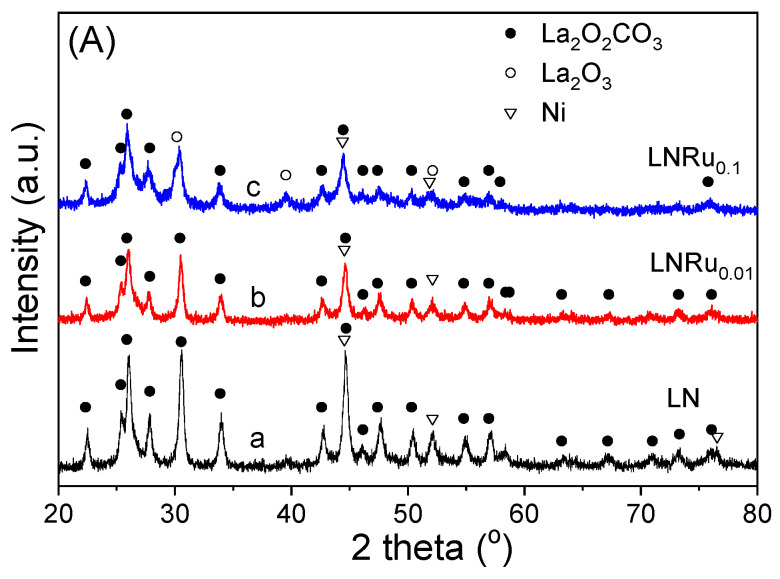

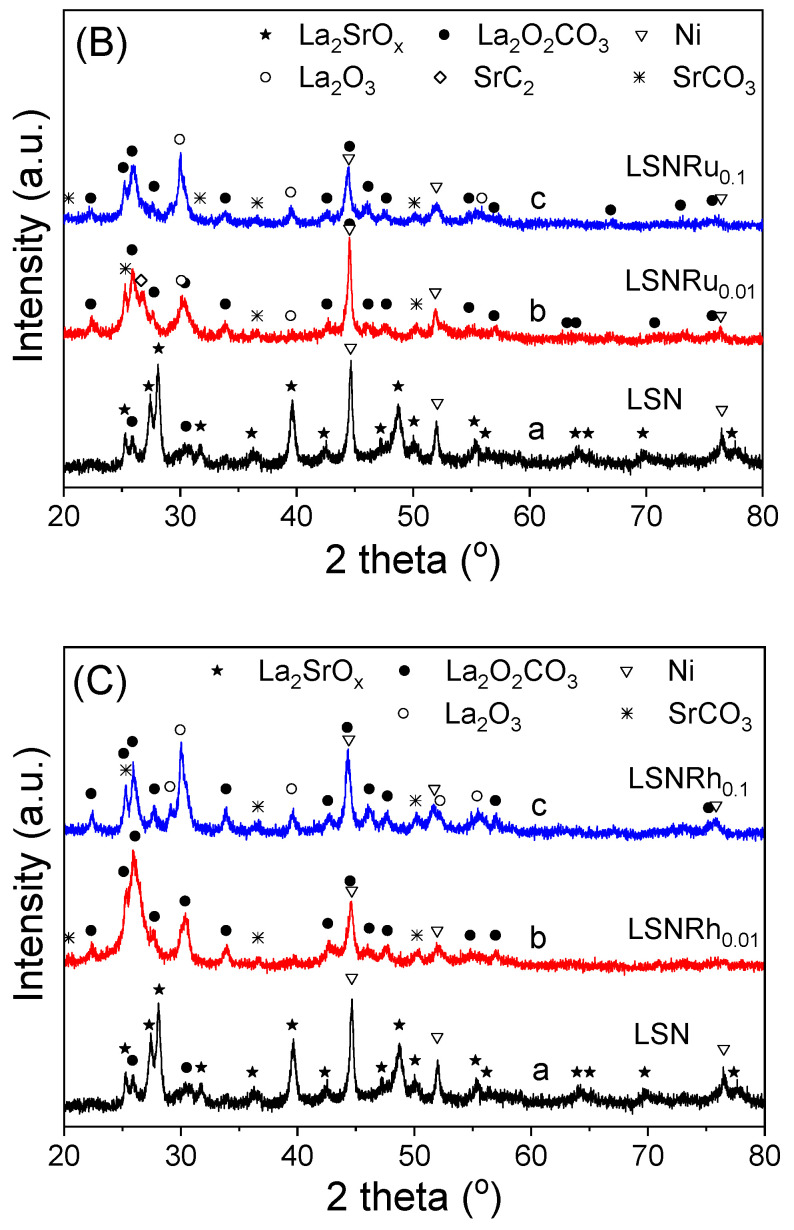
X-ray diffraction patterns obtained for the (**A**) LNRu_x_, (**B**) LSNRu_x_, and (**C**) LSNRh_x_ catalyst samples following exposure to reaction conditions.

The XRD profiles of the used catalysts are presented in Figure 6 and the various phases identified for each sample are listed in Table 1. It was observed that the XRD pattern of LN (Figure 6A, trace a) consisted of peaks attributed to hexagonal La_2_O_2_CO_3_ (JCPDS Card No. 37-804) and cubic Ni (JCPDS Card No. 1-1260). Qualitatively similar results were obtained for the used LNRu_0.01_ (trace b) and LNRu_0.1_ (trace c) catalysts. In the latter case, reflections attributable to hexagonal La_2_O_3_ (JCPDS Card No. 5-602) could also be discerned. This implies that, under the reducing atmosphere existing under reaction conditions, the parent LaNiO_3_ perovskite is in situ transformed into metallic Ni and La_2_O_3_. Because of the high affinity between La_2_O_3_ and CO_2_, lanthanum oxycarbonate (La_2_O_2_CO_3_) is formed according to [66]:

La_2_O_3_ + CO_2_ → La_2_O_2_CO_3_ (7)

The Ni^0^ crystallites, which provide the active sites for propane reforming, were well dispersed and interacted strongly with the La_2_O_3_/La_2_O_2_CO_3_ support, thereby inhibiting metal sintering [3]. The absence of peaks attributable to Ru provides evidence that the exsolved noble metal was also well dispersed on the catalyst surface. As shown in Figure 6B, the XRD pattern of the used LSN sample (trace a) was dominated by reflections attributed to the La_2_SrO*_x_* structure (JCPDS Card No. 42-343) as well as hexagonal La_2_O_3_ and cubic Ni. Interestingly, the La_2_SrO*_x_* phase was absent in the diffraction profiles of the used LSNRu_0.01_ (trace b) and LSNRu_0.1_ (trace c) catalysts that consisted mainly of La_2_O_2_CO_3_, Ni as well as orthorhombic SrCO_3_ (JCPDS Card No. 5-418), La_2_O_3_, and cubic SrC_2_ (JCPDS Card No. 1-1022). This indicates that the presence of Ru in the perovskite structure facilitates the conversion of La_2_SrO*_x_* to La_2_O_3_ and SrO, which may interact with CO_2_ to form La_2_O_2_CO_3_ (Equation (7)) and SrCO_3_:

SrO + CO_2_ → SrCO_3_ (8)

Qualitatively similar results were obtained for the used LSNRh*_x_* catalyst samples (Figure 6C). In particular, the presence of Rh leads in the development of the La_2_O_2_CO_3_, Ni^0^, SrCO_3_, and La_2_O_3_ phases under reaction conditions. As a general trend, the presence of carbonate phases in the used LSNRu*_x_* and LSNRh*_x_* catalyst samples seems to be related to improved catalytic performance for the title reaction. For example, LSN, which is the least active among the samples investigated, was the only one that contained small amounts of La_2_O_2_CO_3_ after exposure to the reaction conditions (Figure 6). The cell parameters estimated for the La_2_O_2_CO_3_ structure detected in the used samples are presented in Table S1. It was observed that in the case of the LSNRu_x_ and LSNRh_x_ catalysts, the *a* cell parameter of La_2_O_2_CO_3_ decreased and the *c* cell parameter increased, compared to the used LSN sample. The EDS mapping analysis performed for the used LSNRh_0.1_ sample (Figure S2) showed intense signals for the carbon and La elements originating from La_2_O_2_CO_3_. It is important to note that no XRD peaks attributable to graphite were detected over the used catalyst samples, indicating that the amount of accumulated carbon deposited was relatively small.

### 3.4. Long-Term Stability Tests

**Figure 7:**
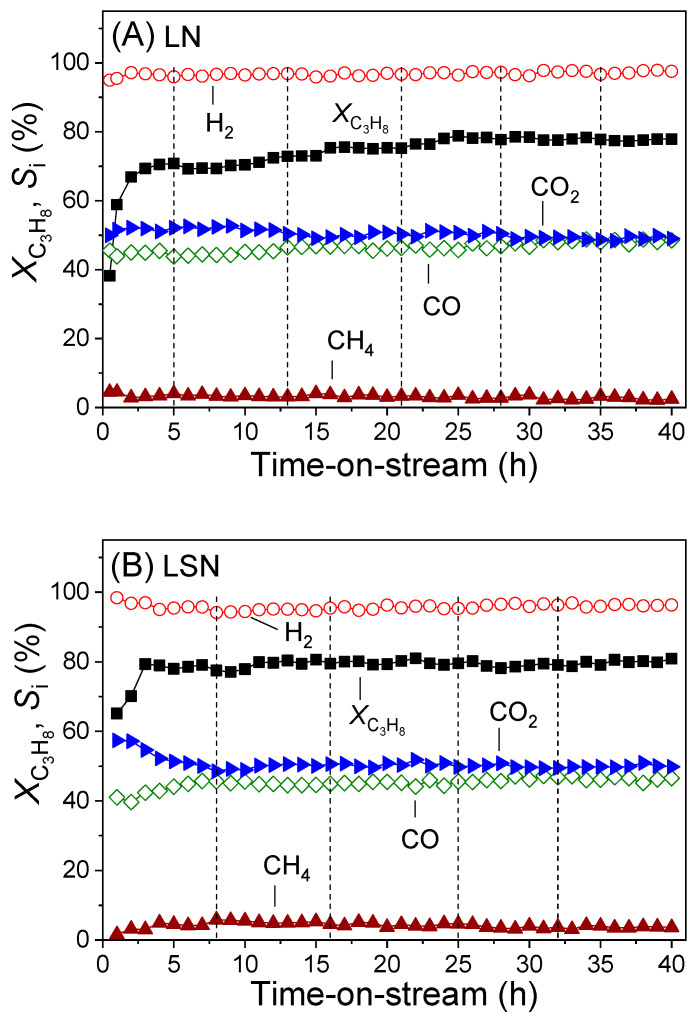

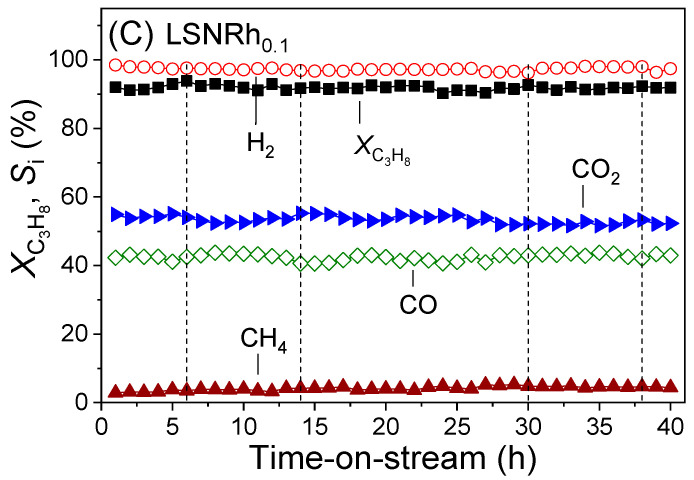
Long-term stability tests obtained at *T* = 600 °C over the (**A**) LN, (**B**) LSN, and (**C**) LSNRh_0.1_ catalysts. Experimental conditions: same as in Figure 3. Vertical lines indicate shutting down of the system overnight, where the catalyst was kept at room temperature under He flow.

The stability of representative catalyst samples derived from LN, LSN, and LSNRh_0.1_ was studied at 600 °C for 40 h. The results obtained are presented in Figure 7, where the conversion of propane (*X*_C3H8_) and the selectivities toward H_2_, CO, CO_2_, and CH_4_ are plotted as functions of time-on-stream. Dashed vertical lines indicate shutting down of the system overnight, where the catalysts were kept at room temperature under He flow. It was observed that the conversion of propane over the LN sample increased substantially during the first three hours-on-stream from an initial value of 38% to ca. 70% (Figure 7A). Prolonged exposure to the reaction mixture resulted in a further increase of *X*_C3H8_, which stabilized at ca. 77% after about 25 hours. This behavior can be attributed to the progressive reduction of the as-prepared LaNiO_3_ perovskite (Figure 1A) to the catalytically active Ni^0^ and La_2_O_2_CO_3_ species (Figure 6A), in agreement with the results of previous studies [31,33,63]. Selectivities to reaction products also varied during the first few hours-on-stream and then remained practically stable. Selectivity toward H_2_ exceeded 97%, whereas *S*_CO_ and *S*_CO2_ had values around 50% (Figure 7A). 

The performance of the LSN catalyst (Figure 7B) was qualitatively similar to that of LN (i.e., *X*_C3H8_ increased during the first few hours-on-stream and then remained stable at ca. 80% throughout the run). The selectivity toward H_2_ remained practically constant at ca. 96%. It is of interest to note that the in situ activation of the LSN sample and the stabilization of catalytic performance (Figure 7B) occurred after a shorter time of exposure to the reaction mixture compared to the LN sample (Figure 7A). This can be attributed to the ability of the Sr dopant to decrease the reduction temperature of the parent material [81] and to facilitate the formation of La_2_O_2_CO_3_ according to:

La_2_O_3_ + SrCO_3_ → La_2_O_2_CO_3_ + SrO (9)

Finally, the LSNRh_0.1_ catalyst (Figure 7C) exhibited excellent stability and was characterized by the highest values of both propane conversion (*X*_C3H8_ = 92%) and selectivity toward H_2_ (*S*_H2_ = 97%) compared to the LN and LSN samples. The fact that *X*_C3H8_ and *S_i_* acquired stable values at relatively very short time periods indicates that the presence of Rh in the structure of the parent material has a beneficial effect on the reduction processes that lead to the in situ formation of the catalytically active Ni^0^ and La_2_O_2_CO_3_ phases. Regarding the higher activity and H_2_ selectivity of the LSNRh_0.1_ catalyst compared to LN and LSN, these can be attributed to the presence of well-dispersed Rh crystallites on the catalyst surface, which act synergistically with Ni^0^ species [72]. 

The excellent stability of the LN, LSN, and LSNRh_0.1_ catalysts (Figure 7) can be related to the formation of La_2_O_2_CO_3_ under reaction conditions. It is well known that this species can prevent catalyst deactivation by reacting with carbon deposits according to [82,83]:

La_2_O_2_CO_3_ + C ⇄ La_2_O_3_ + 2 CO (10)

Similar results have been reported for conventionally prepared Ni/La_2_O_3_ catalysts, which transform into Ni/La_2_O_3_/La_2_O_2_CO_3_ under reduction conditions [84]. It may also be noted that pre-synthesized La_2_O_2_CO_3_ either alone [85,86] or in combination with Al_2_O_3_ [87] has also been used as catalyst supports for reforming reactions. Compared to these materials, the perovskite-derived catalysts have the advantage of comprising very well dispersed and strongly bound metal crystallites on the support, rendering them more active and resistant against carbon deposition. 

### 3.5. Carbon Accumulation on the Catalyst Surface

**Figure 8:**
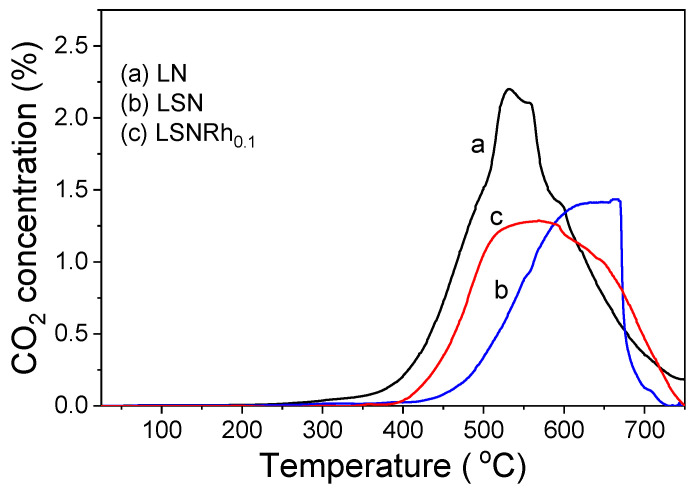
Temperature-programmed oxidation (TPO) profiles obtained for the indicated catalysts following exposure to the long-term stability tests shown in Figure 7.

After the completion of the stability tests presented in Figure 7, TPO experiments were carried out to quantify the amount of reactive carbon-containing species that accumulated on the catalyst surfaces. The results obtained are presented in Figure 8, where the CO_2_ concentration at the reactor effluent is plotted as a function of temperature. It was observed that the TPO profile of the LN-derived catalyst is characterized by an intense peak centered at 530 °C and two shoulders at ca. 560 and 600 °C (trace a). The features appearing at temperatures lower than ca. 550 °C can be attributed to carbon species with amorphous filamentous morphology (C*α*) characterized by a higher reactivity due to structural disorder [88]. The high-temperature shoulder at ca. 600 °C can be assigned to the oxidation of graphite-like carbonaceous species, C*_β_*, which are produced from C_α_ and are more difficult to remove from the catalyst surface ([88] and refs. therein). The amount of accumulated carbon on the LN catalyst was calculated from the area below the TPO curve and was found to be 57 mg C g_cat_^−1^.

The TPO profile of the LSN catalyst consists of a peak with a broad maximum located above ca. 600 °C (trace b). The amount of the accumulated carbon (35 mg C g_cat_^−1^) was much lower than that obtained for the LN sample, indicating that the co-existence of La and Sr at the A-sites of the parent perovskite material decreased the tendency of the derived catalyst toward carbon deposition. As discussed above, this can be related to the ability of SrCO_3_ to facilitate the formation of La_2_O_2_CO_3_ under reaction conditions. Finally, the LSNRh_0.1_ catalyst presented a TPO peak with a broad maximum located at ca. 500–650 °C (trace c), which corresponded to 42.6 mg C g_cat_^−1^. Although the amount of CO_2_ produced was higher than that obtained for LSN (35 mg C g_cat_^−1^), the conversion of propane was also considerably higher (i.e., 92% for LSNRh_0.1_ vs. 80% for LSN (Figure 7)). In addition, the CO_2_ peak started evolving at lower temperatures for the LSNRh_0.1_ sample, indicating that the presence of Rh resulted in the accumulation of filamentous carbonate species, which can be more easily oxidized. This can be attributed to the Rh-induced enhancement of the reducibility of Ni, the inhibition of the sintering of the metallic particles, and the promotion of the gasification rate of carbon deposits due to Ni–Rh interactions [89,90,91]. 

**Figure 9:**
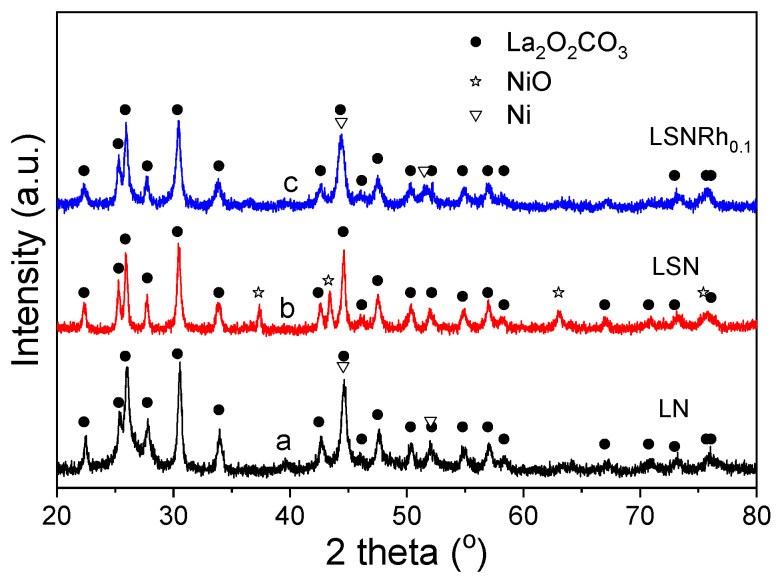
X-ray diffraction patterns of (**a**) LN, (**b**) LSN, and (**c**) LSNRh_0.1_ samples obtained after the TPO experiments presented in Figure 8.

After the TPO measurements, the used samples were characterized with XRD, and the results obtained are shown in Figure 9. It was observed that the dominant phase in the XRD patterns of all three samples, namely LN (trace a), LSN (trace b), and LSNRh_0.1_ (trace c), was La_2_O_2_CO_3_. Nickel was clearly oxidized toward NiO only in the case of LSN, whereas it mainly existed in its metallic phase in the cases of the LN and LSNRh_0.1_ samples. The TPO did not result in the reappearance of the initial perovskite structures. In addition, the La_2_O_2_CO_3_ phase did not decompose, implying that the CO_2_ that evolved during TPO should be mainly attributed to the oxidation of accumulated carbon and not to the decomposition of carbonate phases such as La_2_O_2_CO_3_. This is rather expected since temperatures higher than ca. 900 °C are typically necessary for the decomposition of the La_2_O_2_CO_3_ phase [81]. However, the possibility that the TPO peaks presented in Figure 8 are, at least in part, due to the elimination of CO_2_ from surface oxycarbonates and/or strongly adsorbed CO_2_ cannot be excluded [66,92]. Further investigation of this issue is beyond the scope of the present work and will be the subject of our future studies.

## 4. Conclusions

The effects of partial substitution of La by Sr and of Ni by Ru or Rh in the LaNiO_3_ structure were investigated in an attempt to systematically study the effects of the nature and composition of the A- and B-sites of the perovskite-derived catalysts for the propane steam reforming (PSR) reaction. The physicochemical characteristics of the as-prepared LaNiO_3_ (LN), La_0.8_Sr_0.2_NiO_3_ (LSN), and noble metal-substituted LNM*_x_* and LSNM*_x_* perovskites (M = Ru or Rh; *x* = 0.01 or 0.1) as well as the used catalysts obtained following exposure to reaction conditions were studied by employing the BET method and the XRD technique. Results obtained can be summarized as follows:

1. Incorporation of noble metals in the matrix of LN and LSN perovskites resulted in an increase in the specific surface area (SSA), a shift of the XRD lines toward lower angles, and a decrease in the mean primary crystallite size of the materials. These modifications of the physicochemical characteristics of the perovskites, which are more pronounced for samples with higher noble metal content, have been attributed to the distortion of the perovskite structure induced by the incorporation of Ru or Rh in the matrix;
2. Exposure of the perovskite samples to PSR reaction conditions resulted in the destruction of the perovskite structure and the development of new phases, accompanied by a considerable increase in the specific surface areas of the materials. Specifically, the in situ reduction of the parent perovskites resulted in the exsolution of Ni (as well as Rh or Ru) and the formation of well-dispersed metal nanoparticles on the resulting support, which consisted mainly of La_2_O_2_CO_3_ produced from the interaction of La_2_O_3_ with CO_2_. The main phases detected with XRD for the LNRu*_x_*-derived samples included metallic Ni and La_2_O_2_CO_3_ whereas the LNSRu*_x_* and LNSRh*_x_*-derived catalysts also contained La_2_SrO*_x_*, La_2_O_3_, and SrCO_3_. The presence of noble metals in the derived catalysts was confirmed by SEM/EDS measurements;
3. The LN-derived catalyst exhibited higher activity compared to LSN, and its performance for the title reaction did not change appreciably following partial substitution of Ru in the B-sites of the perovskite. In contrast, incorporation of Ru and, especially, Rh in the perovskite matrix resulted in the development of catalysts with significantly improved catalytic performance, which increased with an increase in the noble metal content;
4. Results of long-term stability test obtained at 600 °C using the LN, LSN, and LSNRh_0.1_ samples showed that all catalysts were characterized by high stability for 40 hours-on-stream, which was reflected in the accumulation of relatively small amounts of carbon deposits on the catalyst surfaces. This has been attributed to the in situ formation of La_2_O_2_CO_3_ under reaction conditions, which facilitates the oxidation of accumulated carbon, thereby preventing catalyst deactivation; and
5. Best results were obtained for the LSNRh_0.1_-derived catalyst, which was characterized by high activity (*X*_C3H8_ = 92%) and selectivity toward H_2_ (*S*_H2_ = 97%) at 600 °C as well as excellent stability for 40 hours-on-stream. This has been attributed to the presence of well dispersed Rh crystallites on the catalyst surface, which act synergistically with Ni^0^ species.

## Figures and Tables

**Table 1:** Notation, nominal composition, BET specific surface area, and main phases detected with XRD for the as-prepared (fresh) perovskites and the used catalysts.

Notation	Nominal Formula	Specific Surface Area (m^2^ g^−1^)		Phases Detected with XRD	
		Fresh	Used	Fresh	Used
LN	LaNiO_3_	3	42	LaNiO_3_ (rhombohedral, rh)	La_2_O_2_CO_3_ Ni
LSN	La_0.8_Sr_0.2_NiO_3_	5	18	Sr_0.5_La_1.5_NiO_4_ NiO	La_2_SrO_x_ La_2_O_2_CO_3_ Ni
LNRu_0.01_	LaNi_0.99_Ru_0.01_O_3_	4	56	LaNiO_3_ (rh)	La_2_O_2_CO_3_ Ni
LNRu_0.1_	LaNi_0.9_Ru_0.1_O_3_	6	71	LaNiO_3_ (cubic)	La_2_O_2_CO_3_ La_2_O_3_ Ni
LSNRu_0.01_	La_0.8_Sr_0.2_Ni_0.99_Ru_0.01_O_3_	6	55	Sr_0.5_La_1.5_NiO_4_ LaNiO_3_ (rh) SrNiO_3_ NiO	La_2_O_2_CO_3_ SrCO_3_ SrC_2_ Ni
LSNRu_0.1_	La_0.8_Sr_0.2_Ni_0.9_Ru_0.1_O_3_	8	59	Sr_0.5_La_1.5_NiO_4_ LaNiO_3_ (rh) NiO	La_2_O_2_CO_3_ SrCO_3_ La_2_O_3_ Ni
LSNRh_0.01_	La_0.8_Sr_0.2_Ni_0.99_Rh_0.01_O_3_	6	66	Sr_0.5_La_1.5_NiO_4_ NiO	La_2_O_2_CO_3_ SrCO_3_ Ni
LSNRh_0.1_	La_0.8_Sr_0.2_Ni_0.9_Rh_0.1_O_3_	6	29	Sr_0.5_La_1.5_NiO_4_ NiO	La_2_O_2_CO_3_ SrCO_3_ La_2_O_3_ Ni

## Data Availability

Not applicable.

## Supplementary Materials

The following are available online at https://www.mdpi.com/article/10.3390/nano11081931/s1. Figure S1. SEM image (A), EDS mapping results showing the distribution of (B) La, (C) Sr, (D) Ni, (E) Rh and (F) La (green spots), Ni (blue spots) and Rh (red spots) elements and corresponding EDS spectrum (G) over the as-prepared LSNRh_0.1_ perovskite sample; Figure S2. SEM image (A), EDS mapping results showing the distribution of (B) Carbon, (C) La, (D) Sr, (E) Ni and (F) Rh elements and corresponding EDS spectrum (G) over the “used” LSNRh0.1 perovskite sample; Table S1: Cell parameters of the crystalline phases detected by XRD for the as-prepared perovskite samples and the derived (used) catalysts.
