# Supplementary material for: Propane Steam Reforming over Catalysts Derived from Noble Metal (Ru, Rh)-Substituted LaNiO3 and La0.8Sr0.2NiO3 Perovskite Precursors
Source: Nanomaterials (Basel). 2021 Jul 27;11(8):1931. doi: 10.3390/nano11081931 (PMC8401020; doi:10.3390/nano11081931)
Supplement: Supplementary file 1 [file nanomaterials-11-01931-s001.zip › nanomaterials-1298801-supplementary.pdf]

Supplementary Materials

Propane Steam Reforming over Catalysts Derived from Noble Metal (Ru, Rh)-Substituted $LaNiO_3$ and $La_{0.8}Sr_{0.2}NiO_3$ Perovskite Precursors

Theodora Ramantani, Georgios Bampos, Andreas Vavatsikos, Georgios Vatskalis and Dimitris I. Kondarides *

Department of Chemical Engineering, University of Patras, GR-26504 Patras, Greece;
ramantani@chemeng.upatras.gr (T.R.); geoba@chemeng.upatras.gr (G.B.); up1019056@upnet.gr (A.V.);
up1047645@upnet.gr (G.V.)
* Correspondence: dimi@chemeng.upatras.gr; Tel.: +30 2610969527; Fax: +30 2610991527

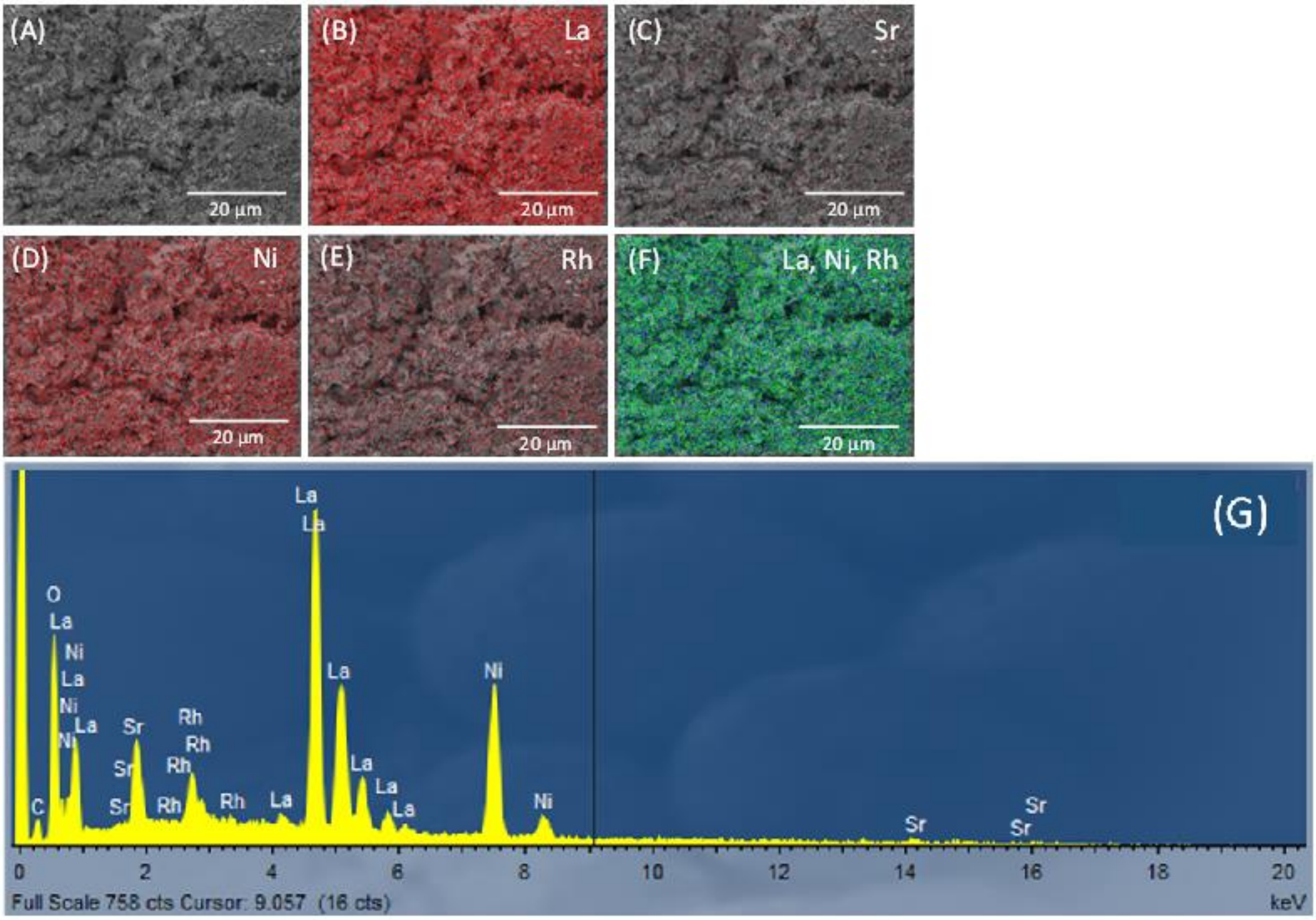

Figure S1. SEM image (A), EDS mapping results showing the distribution of (B) La, (C) Sr, (D) Ni, (E) Rh and (F) La (green spots), Ni (blue spots) and Rh (red spots) elements and corresponding EDS spectrum (G) over the as-prepared $LSNRh_{0.1}$ perovskite sample.

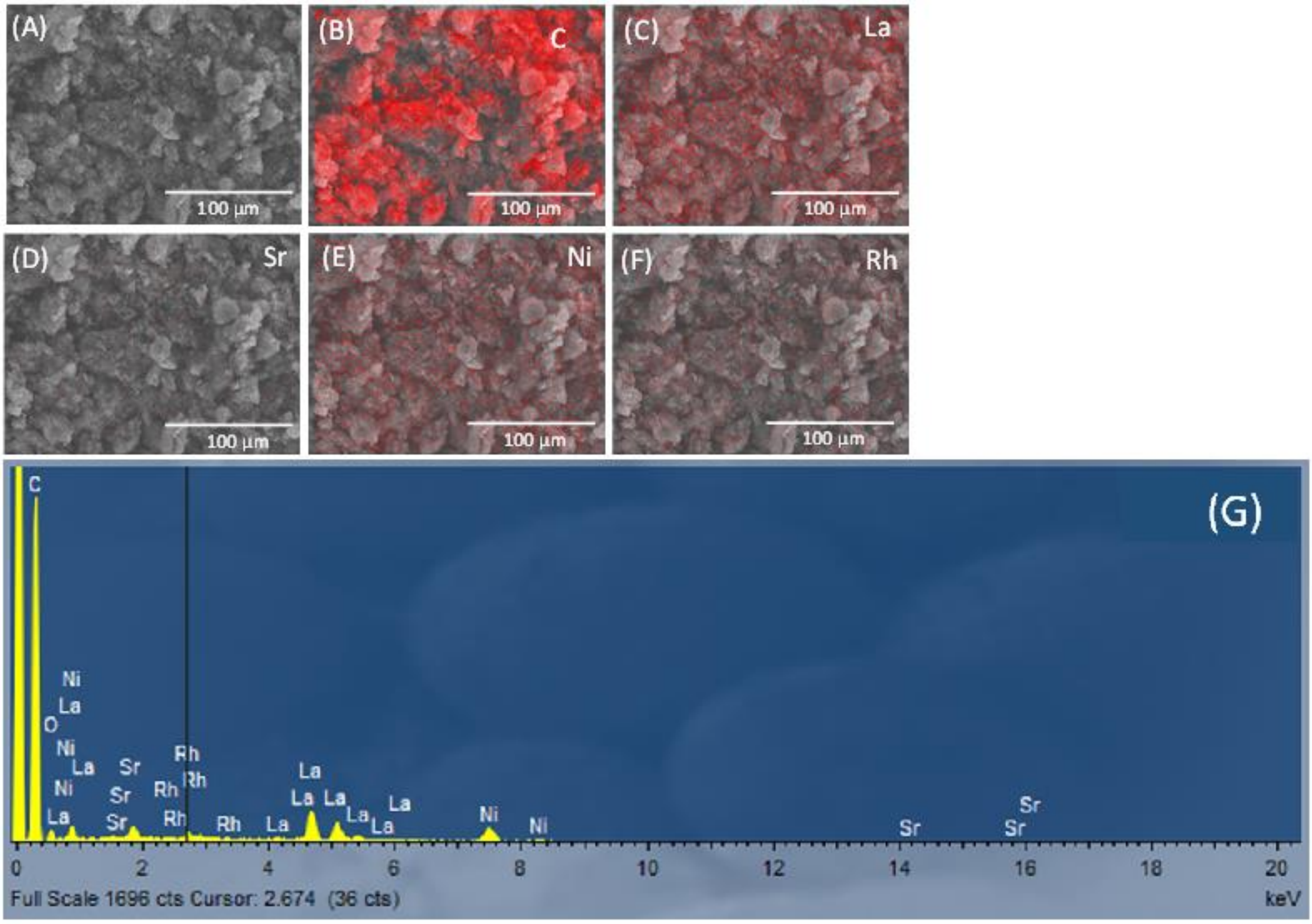

Figure S2. SEM image (A), EDS mapping results showing the distribution of (B) Carbon, (C) La, (D) Sr, (E) Ni and (F) Rh elements and corresponding EDS spectrum (G) over the "used" $LSNRh_{0.1}$ perovskite sample.

Table S1. Cell parameters of the crystalline phases detected by XRD for the as-prepared perovskite samples and the derived (used) catalysts.

Notation	Phase detected with XRD	Cell Parameters		Phase detected with XRD	Cell Parameters	
	Fresh	Fresh		Used	Used	
LN	$LaNiO_3$ rhombohedral	*a* = 5.686 Å	-	$La_2O_2CO_3$, hexagonal	*a* = 4.057 Å	*c* = 15.675 Å
LSN	$Sr_{0.5}La_{1.5}NiO_4$, tetragonal	*a* = 3.802 Å	*c* = 12.659 Å	$La_2O_2CO_3$, hexagonal	*a* = 4.068 Å	*c* =15.882 Å
$LNRu_{0.01}$	$LaNiO_3$ rhombohedral	*a* = 5.686 Å	-	$La_2O_2CO_3$, hexagonal	*a* = 4.055 Å	*c* = 16.014 Å
$LNRu_{0.1}$	$LaNiO_3$ cubic	*a* = 3.862 Å	-	$La_2O_2CO_3$, hexagonal	*a* = 4.066 Å	*c* = 16.071 Å
$LSNRu_{0.01}$	$Sr_{0.5}La_{1.5}NiO_4$, tetragonal	*a* = 3.832 Å	*c* = 12.539 Å	$La_2O_2CO_3$, hexagonal	*a* = 4.063 Å	*c* = 16.288 Å
$LSNRu_{0.1}$	$Sr_{0.5}La_{1.5}NiO_4$, tetragonal	*a* = 3.822 Å	*c* = 12.578 Å	$La_2O_2CO_3$, hexagonal	*a* = 4.053 Å	*c* = 16.170 Å
$LSNRh_{0.01}$	$Sr_{0.5}La_{1.5}NiO_4$, tetragonal	*a* = 3.822 Å	*c* = 12.656 Å	$La_2O_2CO_3$, hexagonal	*a* = 4.063 Å	*c* = 16.009 Å
$LSNRh_{0.1}$	$Sr_{0.5}La_{1.5}NiO_4$, tetragonal	*a* = 3.822 Å	*c* = 12.578 Å	$La_2O_2CO_3$, hexagonal	*a* = 4.049 Å	*c* = 16.003 Å
